# Comparative Five-Year Surgical Outcomes of Open-Door versus French-Door Laminoplasty in Multilevel Cervical Spondylotic Myelopathy

**DOI:** 10.1155/2020/8853733

**Published:** 2020-12-07

**Authors:** Guoliang Chen, Xizhe Liu, Ensi Zhao, Ningning Chen, Fuxin Wei, Shaoyu Liu

**Affiliations:** ^1^Department of Orthopedic Surgery, The Seventh Affiliated Hospital, Sun Yat-sen University, Shenzhen, China; ^2^Guangdong Provincial Key Laboratory of Orthopaedics and Traumatology, Orthopaedic Research Institute, Department of Spine Surgery, The First Affiliated Hospital, Sun Yat-sen University, Guangzhou, China

## Abstract

**Objective:**

To compare the five-year surgical outcomes between Open-Door laminoplasty (ODL) and French-Door laminoplasty (FDL) in the management of multilevel cervical spondylotic myelopathy (MCSM).

**Methods:**

Sixty patients with MCSM, who were operated by ODL or FDL, were included in this study and followed up for at least 5 years. The average follow-up period was 69.2 ± 3.2 months. The modified Japanese Orthopaedic Association (mJOA) score and radiological assessments including the Cobb angle and cervical range of motion (ROM) were evaluated and compared before surgery and at the final follow-up. The incidence of postoperative complications and medical costs were also compared.

**Results:**

Both ODL and FDL groups achieved significant improvements of the mJOA score in postoperative 5 years; the average recovery rate (RR) of the mJOA score in the ODL and FDL groups was 72.14 ± 6.97% and 69.53 ± 7.51%, respectively. No statistically significant differences regarding the pre- and postoperative mJOA score, the RR of the mJOA score, the loss and the loss rate of the Cobb angle, and the incidence of postoperative complications existed between ODL and FDL. The mean loss and the loss rate of cervical ROM in the FDL group (18.70 ± 8.91°, 41.08 ± 11.17%) were significantly higher than those of the ODL group (13.81 ± 8.62°, 31.47 ± 12.43%) (*P* < 0.05). FDL reduced medical costs more greatly than ODL (33014.37 ± 3424.12 China Yuan versus 82096.62 ± 7093.07 China Yuan, *P* < 0.001).

**Conclusions:**

Both ODL and FDL are effective for MCSM. The 5-year neurological results are similar between the two groups. ODL trends to be superior to FDL in postoperative preservation of cervical ROM while FDL reduced medical costs more greatly.

## 1. Introduction

**Figure 1:**
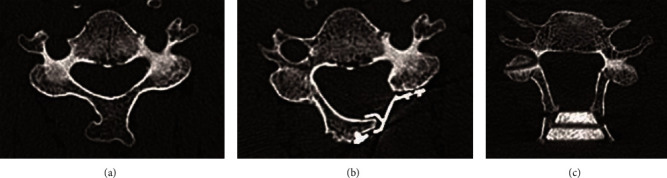
The diagram of laminoplasty: (a) the preoperative cross-sectional CT image of cervical spine; (b) the postoperative cross-sectional CT image of cervical spine with Open-Door laminoplasty; (c) the postoperative cross-sectional CT image of cervical spine with French-Door laminoplasty.

For the multilevel cervical spondylotic myelopathy (MCSM), posterior approaches such as laminoplasty and laminectomy have been recognized as effective methods [1, 2]. However, according to previously published studies, operation-related complications such as cervical kyphosis, segmental instability, and neurological deterioration which were likely due to cervical instability after posterior structures being removed were not uncommon with patients treated with laminectomy [3, 4]. Therefore, laminoplasty is put into application as an alternative to laminectomy, which permits adequate decompression while maintaining mechanical stability and motion of the cervical spine [5, 6]. Laminoplasty is generally performed via either Open-Door Laminoplasty (ODL) or French-Door Laminoplasty (FDL) (Figure 1). With ODL, the spinal canal is opened on one side and hinged on the other, creating an asymmetrical expansion of the canal [5]. FDL involves opening the “door” in the midline, which creates a symmetrical opening of the canal. Although both methods have been reported to show satisfactory short-term clinical outcomes, several complications still existed, such as the loss of cervical range of motion (ROM) and lordosis, postoperative axial symptoms, and C5 palsy, which have been considered the main factors influencing surgical outcomes [7]. In addition, several studies comparing the short-term outcomes of these two approaches have been published [8, 9], but studies on long-term outcomes are still scarce. In order to compare the long-term surgical outcomes, we retrospectively analyzed the five-year effectiveness and postoperative complications of ODL and FDL in the treatment of MCSM in this study.

## 2. Materials and Methods

### 2.1. Study Populations

This study was approved by the Institutional Review Board of our hospital. From March 2011 to March 2014, seventy-five patients with MCSM accepted cervical laminoplasty performed by the same chief surgeon in the study hospital. Patients with history of musculoskeletal trauma, infection, tumor, surgery, or other neurological disorders before the latest follow-up were excluded from this study. Finally, 6 patients in the ODL group and 9 patients in the FDL group were excluded, and a total of 60 patients who were followed for at least 5 years were included in this study. According to the surgical methods, the patients were divided into two groups: ODL group (*n* = 25, 18 male and 7 female) and FDL group (*n* = 35, 23 male and 12 female).

### 2.2. Surgical Techniques

The ODL was performed according to Hirabayashi et al.'s method with some modifications [5]. The laminae were exposed through a midline incision followed by dissection of the bilateral paracervical muscles (the attachments of semispinalis in C2 and C7 were preserved). A rongeur was used to make a gutter at the junction of the lamina and facet joint, and the ventral cortex of the lamina was perforated. Another gutter was made on the opposite side as a hinge, and the laminar door was lifted and fixed in the expanded position with a miniplate.

The FDL was performed in compliance with Kurokawa and Tanaka's method with some modifications [10]. After detaching bilateral paravertebral muscles from the spinous (the method was in line with that of the ODL group), all spinous processes within the surgical range were removed. The center of the laminae was cut using a fretsaw. Bilateral gutters were created as hinges by a rongeur at the border of the laminae and facets. After the halves of the laminae were elevated, a sizeable hydroxyapatite spacer was tied to bridge the bilateral edges of the laminae and fixed with wires.

All patients were allowed to sit up with a soft neck collar and to stand and walk on postoperative Day 1. Removal of the soft collar was allowed 1 week after surgery. All patients were then encouraged to start range of motion and isometric muscle strengthening exercises of the neck as early as possible.

### 2.3. Clinical Assessments

The preoperative symptom duration, operative time, blood loss, and medical costs of the two surgical methods were recorded, respectively. Complications such as incision infection, axial symptoms, and C5 palsy were also recorded at the final follow-up.

Neurological function was evaluated using the modified Japanese Orthopaedic Association (mJOA) scoring system at the final follow-up. The recovery rate (RR) of the mJOA score (%) was calculated using the following formula [11]:

(1) $$\begin{array}{c} \text{RR of mJOA score}=\frac{\left(\text{postoperative mJOA score}–\text{preoperative mJOA score}\right)}{\left(17–\text{preoperative mJOA score}\right)}\times 100\%. \end{array}$$

### 2.4. Radiological Measurements

**Figure 2:**
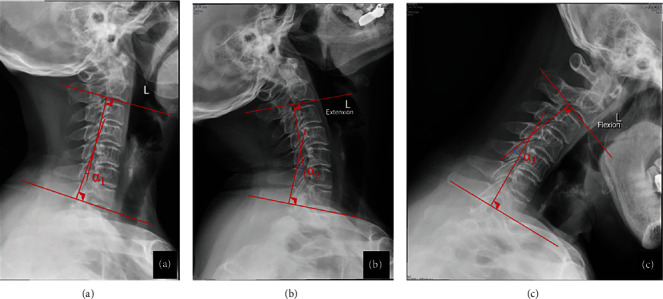
Radiological measurements: (a) the measurement of Cobb angle (*α*_1_) on the neutral position lateral X-ray; (b, c) the measurement of cervical range of motion (ROM) on the extension and flexion lateral X-ray, ROM = *α*_2_+*α*_3_.

Anteroposterior, lateral, and extension-flexion radiographs were conducted before surgery and at the final follow-up. The Cobb angle and cervical ROM were measured using lateral radiographs and dynamic lateral radiographs, respectively. All patients' imaging was assessed by two researchers independently and repeated three times (Figure 2).

### 2.5. Statistics Analysis

Data was statistically analyzed using GraphPad Prism 8.3.0 software and expressed as the mean ± standard deviation. The data including symptom duration, age at surgery, operative time, blood loss, treatment expense, mJOA score, Cobb angle, cervical ROM, RR of the mJOA score, the loss and the loss rate of the Cobb angle, and the loss and the loss rate of cervical ROM were compared between the ODL and FDL groups by the unpaired *t*-test. The mJOA score, Cobb angle, and cervical ROM of each group were compared between preoperative and final follow-up by a paired-sample *t*-test, respectively. The incidence of incision infection, axial symptoms, and C5 palsy were compared between the two groups by a chi-squared test. *P* value < 0.05 indicated a significant difference.

## 3. Results and Discussion

### 3.1. Results

#### 3.1.1. Baseline Data

For the ODL group, the mean preoperative symptom duration was 32.18 ± 48.03 months, mean age at surgery was 57.76 ± 9.52 years, mean operative time was 139.72 ± 32.96 mins, mean blood loss was 136.00 ± 56.05 ml, and mean treatment expense was 82096.62 ± 7093.07 China Yuan. For the FDL group, the mean preoperative symptom duration was 32.92 ± 53.32 months, mean age at surgery was 54.71 ± 12.15 years, mean operative time was 151.29 ± 33.66 mins, mean blood loss was 164.14 ± 95.35 ml, and mean treatment expense was 33014.37 ± 3424.12 China Yuan. As for baseline data, there were no significant differences between the two groups besides the treatment expense (Table 1).

### 3.2. Neurological Assessments

Both postoperative 5-year mJOA scores of the two groups improved significantly compared with those before surgery. No cases were complicated by neurological deterioration. The average mJOA score of the ODL group was significantly improved from preoperative 10.32 ± 1.60 points to 15.12 ± 0.63 points at the final follow-up (*P* < 0.001). The preoperative average mJOA score of the FDL group was 10.33 ± 1.73 points and improved to 15.03 ± 0.53 points at the final follow-up (*P* < 0.001). The average RR of the mJOA score in the ODL and FDL groups at the final follow-up was 72.14 ± 6.97% and 69.53 ± 7.51%, respectively. No significant difference of pre- and postoperative mJOA scores and the RR of the mJOA score was found between the two groups (*P* > 0.05) (Table 2).

### 3.3. Radiographic Outcomes

The mean Cobb angle reduced significantly from preoperative 15.98 ± 8.94° to 11.74 ± 6.59° at the last follow-up in the ODL group (*P* < 0.001) while it reduced significantly from preoperative 16.48 ± 9.33° to 12.30 ± 6.70° in the FDL group (*P* < 0.001). The mean loss of the Cobb angle was 4.18 ± 3.92° and 4.18 ± 2.65° in the ODL and FDL groups, respectively. The mean loss rate of the Cobb angle was 25.42% ± 12.89% and 27.16 ± 12.35% in the ODL and FDL groups, respectively. The difference of pre- and postoperative Cobb angles and the loss and the loss rate of the Cobb angle between ODL and FDL was not significant (*P* > 0.05).

For the ODL group, the cervical ROM at the final follow-up decreased significantly from 42.18 ± 12.32° to 28.31 ± 8.09° (*P* < 0.001), and it also significantly decreased from 44.41 ± 13.27° to 25.61 ± 7.77° in the FDL group (*P* < 0.001). The mean loss of cervical ROM was 13.87 ± 8.62° and 18.80 ± 9.02° in the ODL and FDL groups, while the mean loss rate of ROM was 31.47 ± 12.43% and 41.08 ± 11.17%, respectively. Between the two groups, no statistically significant differences were observed in terms of cervical ROM that were measured preoperatively and at the final follow-up (*P* > 0.05). However, the loss and the loss rate of cervical ROM in the ODL group were statistically lower than those of the FDL group at the last follow-up (*P* < 0.05) (Table 2).

### 3.4. Postoperative Complications

Two cases of incision infection, three cases of axial symptoms, and one case of C5 palsy were documented in the ODL group, while one case of incision infection, two cases of axial symptoms, and one case of C5 palsy were recorded in the FDL group. No other complications such as failure of implants and reclosure of lamina were observed in both groups. There were no significant differences regarding the incidence of incision infection, axial symptoms, and C5 palsy between the two groups (Table 2).

All the incision infections were cured after oral antibiotics were given for two weeks, except one case which was performed with debridement and resutured. All the patients diagnosed with axial symptoms and C5 palsy recovered spontaneously within 6 months after surgery.

## 4. Discussion

In this study, 60 patients with MCSM who underwent ODL or FDL were included. By comparing the neurological outcomes, radiological outcomes, and incidence of complications after ODL and FDL, we found that both two methods achieved satisfactory surgical outcomes, but ODL achieved better preservation of ROM, while FDL reduced medical costs more greatly.

Current studies demonstrated that the main pathogenesis of spinal cord impairment in CSM was the chronic compression and the ischemia and neuroinflammation secondary to compression [12, 13]. The surgical procedures achieve improvement of neurological function by decompressing and increasing the perfusion of the spinal cord [14]. To our knowledge, laminoplasty which decompresses the spinal cord by enlarging the spinal canal has been recognized as one of the most important surgical practices to treat MCSM and achieved satisfactory clinical outcomes [2, 8]. In this study, the average RR of the mJOA score in the ODL and FDL groups at the five-year follow-up was 72.14 ± 6.97% and 69.53 ± 7.51%, respectively, which were in accordance with that of previous studies about the short-term follow-up [8, 15]. The five-year neurological outcome was almost the same with both ODL and FDL and in line with the short-term neurological outcome of previous studies. This indicated that both ODL and FDL were the effective methods for MCSM and the neurological improvement could maintain until postoperative 5 years.

One of the purposes of cervical laminoplasty is to preserve cervical ROM after decompression [16]. In this study, the cervical ROM of both ODL and FDL groups decreased significantly after laminoplasty, which was consistent with the previous studies [17, 18]. However, the average loss and the loss rate of cervical ROM in the ODL group were significantly lower than those in the FDL group; this result was contrary to the study conducted by Nakashima et al. [8]. Previous studies hold the opinion that the preservation of cervical ROM could prevent adjacent segment disease and decrease the axial symptoms. But the specific factors affecting the cervical ROM after cervical laminoplasty remain elusive. Some researchers considered that the condition of posterior cervical muscles was an important factor contributing to the change of cervical ROM after laminoplasty [19, 20]. Fujimura and Nishi [21] found that the area of posterior cervical muscles by CT cross-sectional scan was correlated with the changing curve of ROM. Thus, a variety of modified methods that protect the posterior cervical muscles, such as separating the unilateral paravertebral muscles [22] or preserving the attachment points of semispinalis on C2 and C7 [23], have been used and achieved better outcomes of cervical ROM. In this study, the surgical procedures protecting the posterior cervical muscles in ODL and FDL were consistent, but the postoperative cervical ROM decreased significantly in the FDL group than in the ODL group. We speculated that the different methods of laminoplasty might account for the difference in preservation of cervical ROM. The healing of bilateral gutters of FDL might increase the incidence of interlaminar bony fusion; then, the increased bony fusion of FDL constricted the cervical ROM more stringent. However, further studies are warranted to verify this speculation.

It has been reported that the complications following laminoplasty, such as cervical kyphosis, axial symptoms, and C5 palsy, resulted in serious impact on the patient's quality of life postoperatively [24–29]. Current studies reported that factors such as the destruction of the posterior cervical structures, the instability of the facet joint, and the atrophy of the posterior cervical muscles caused by prolonged intraoperative traction were correlated with the postoperative cervical kyphosis and axial symptoms [30] and that C5 palsy is correlated with traction after the spinal cord drifts backward, segmental ischemia, and ischemia-reperfusion injury of the spinal cord [31, 32]. Our results revealed that the cervical alignment was still maintained in lordosis and the incidence of axial symptoms was also lower than that of previous studies [29, 30]; it may benefit from the protection of posterior cervical muscles, especially the semispinalis [23].

In addition to the five-year effectiveness of neurological and radiological outcomes, the significant difference in medical cost between these two methods should cause our concern. The miniplate used in ODL was much more expensive than the hydroxyapatite spacer used in FDL; results of the present study revealed that the long-term results of neurological recovery and cervical alignment were similar between ODL and FDL, but the application of FDL reduced the medical cost greatly. To promote the application of FDL seems to be greatly significant and necessary.

In this retrospective study, there are several limitations that should be noteworthy. Relatively small sample size may result in low credibility. The information bias is easy to occur due to the subjectivity of the mJOA score. To obtain a precise conclusion, a prospective randomized control study with large samples and more objective evaluation indexes is imperative, which should be conducted in the next ten years (clinical Trial No.ChiCTR180001704).

## 5. Conclusions

Both ODL and FDL are effective in treating MCSM. The five-year neurological results are similar between the two groups. ODL trends to be superior to FDL in postoperative preservation of cervical ROM while FDL reduced medical costs more greatly.

## Figures and Tables

**Table 1:** Baseline data of patients.

	ODL	FDL	*P*
Number of patients	25	35	
Male	18	23	0.613
Female	7	12	
Age at surgery (year)	57.76 ± 9.52	54.71 ± 12.15	0.300
Preoperative symptom duration (month)	32.18 ± 48.03	32.92 ± 53.32	0.954
Operative time (min)	139.72 ± 32.96	151.29 ± 33.66	0.190
Blood loss (ml)	136.00 ± 56.05	164.14 ± 95.35	0.192
Medical expense (China Yuan)	82096.62 ± 7093.07	33014.37 ± 3424.12	<0.001^∗^

^∗^Comparison between groups, *P* < 0.05. ODL: Open-Door laminoplasty; FDL: French-Door laminoplasty.

**Table 2:** The results of neurological function, radiographic data, and postoperative complications.

	ODL	FDL	*P*
Case number	25	35	
*mJOA score*			
Preoperative	10.32 ± 1.60	10.33 ± 1.73	0.9845
Final follow-up	15.12 ± 0.63^∗^	15.03 ± 0.53^∗^	0.545
Recovery rate of mJOA score at the final follow-up (%)	72.14 ± 6.97	69.53 ± 7.51	0.177
*Radiographic data*			
Preoperative Cobb angle (°)	16.48 ± 9.33	15.99 ± 8.24	0.8288
Cobb angle at the final follow-up (°)	12.30 ± 6.70^∗^	11.81 ± 6.58^∗^	0.778
Loss of Cobb angle at the final follow-up (°)	4.18 ± 3.92	4.18 ± 2.65	0.9963
Loss rate of Cobb angle at the final follow-up (%)	25.42 ± 12.89	27.16 ± 12.35	0.600
Preoperative cervical ROM (°)	42.18 ± 12.32	44.41 ± 13.27	0.512
Cervical ROM at the final follow-up (°)	28.31 ± 8.09^∗^	25.61 ± 7.77^∗^	0.198
Loss of cervical ROM at the final follow-up (°)	13.81 ± 8.62	18.70 ± 8.91	0.038^∗∗^
Loss rate of cervical ROM at the final follow-up (%)	31.47 ± 12.43	41.08 ± 11.17	0.003^∗∗^
*Postoperative complication (case numbers)*			
Incision infection	2	1	0.565
Axial symptoms	3	2	0.640
C5 palsy	1	1	1.000

^∗^Compared with preoperative, *P* < 0.001; ^∗∗^comparison between two groups, *P* < 0.05. ODL: Open-Door laminoplasty; FDL: French-Door laminoplasty; mJOA score: modified Japanese Orthopaedic Association score; ROM: range of motion.

## Data Availability

The data used to support the findings of this study were collected by authors under license and so cannot be made freely available. Requests for access to these data should be made to the corresponding author.
